# Expression of candidate marker genes of sugar starvation is upregulated in growth-suppressed parthenocarpic cucumber fruit. Novel gene markers for sugar starvation in growth-suppressed cucumber fruit

**DOI:** 10.3389/fpls.2023.1241267

**Published:** 2023-08-18

**Authors:** Akio Tazuke, Tsuguki Kinoshita, Munehiko Asayama

**Affiliations:** College of Agriculture, Ibaraki University, Ibaraki, Japan

**Keywords:** asparagine synthetase, CsSEF1, fruit abortion, fruit growth suppression, RNA-seq

## Abstract

To examine the physiological change in the growth suppression and abortion of parthenocarpic cucumber fruit, the expression of candidate marker genes of sugar starvation in relation to growth activity was examined. Fruits that failed to start exponential growth seemed to eventually abort. Hexose concentration of fruits was low in growth-suppressed fruit and increased in normally growing fruit consistent with the vacuolization. The correlation matrix indicated that the transcript levels of the genes, except CsaV3_6G046050 and CsaV3_5G032930, had a highly significant negative correlation with the relative growth rate in fruit length and had highly significant mutual positive correlations, suggesting that the asparagine synthetase gene, *Cucumis sativus* putative CCCH-type zinc finger protein CsSEF1, *C. sativus* BTB/POZ domain-containing protein At1g63850-like, CsaV3_3G000800, CsaV3_3G041280, and CsaV3_7G032930 are good markers of sugar starvation in cucumber fruit. The expression of candidate marker genes together with the hexose analysis strongly suggests that severe sugar starvation is occurring in growth-suppressed fruit.

## 1 Introduction

Sugar starvation occurs in plant tissues under various stress conditions (Slocombe et al., 2004; Barros et al., 2017; van Rensburg et al., 2019). In *Arabidopsis* and other plants, hexokinase 1 (Moore et al., 2003), SnRK1, (Lu et al., 2007), trehalose 6-phosphate (Fichtner and Lunn, 2021), TOR (Dobrenel et al., 2016; Li et al., 2019; Rodriguez, 2019), mR156 (Buendia-Monreal and Gillmore, 2017) are known to be involved in sugar signaling. On the other hand, asparagine synthase gene is regarded to be a very good marker of sugar starvation (Rolland et al., 2006). This may mean that the expression of the genes related to sugar signaling is not necessarily a good marker of sugar starvation. Also, sugar starvation has been studied mainly in leaves. So, we looked for marker genes of sugar starvation in cucumber fruit in relation to the formation of malformed fruits. Using the subtraction method (Tazuke and Asayama, 2013; Tazuke et al., 2015), we identified three candidate genes that are upregulated by total defoliation including the asparagine synthetase (*AS*) gene,. The function of the remaining two genes remains unclear. However, the expression of *Cucumis sativus* putative CCCH-type zinc finger protein CsSEF1 (*CsSEF1*) and *C. sativus* BTB/POZ domain-containing protein At1g63850-like (*CsFDI1*) was markedly upregulated by total defoliation (Tazuke and Asayama, 2013; Tazuke et al., 2015), prolonged darkness (Tazuke and Asayama, 2013; Tazuke et al., 2015), salinity (Tazuke et al., 2017), removal of the ovary (Tazuke et al., 2017), and fruit harvest (Tazuke and Kinoshita, 2018), strongly suggesting that these genes are good markers of sugar starvation. In a preliminary experiment, the growth cessation of parthenocarpic cucumber fruit was accompanied by upregulation of the *AS*, *CsSEF1*, and *CsFDI1* genes, suggesting the occurrence of sugar starvation during growth cessation or abortion of a fruit (Tazuke, unpublished data). In addition, we isolated five genes by RNA sequencing (RNA-seq) followed by real-time PCR (Tazuke et al., 2019) of total defoliated ‘Tokiwa’; the expression of PREDICTED: *C. sativus* homeobox-leucine zipper protein ATHB-40 (*HB*), PREDICTED: *C. sativus* peptide methionine sulfoxide reductase (*PMS*), PREDICTED: *C. sativus* NAC domain-containing protein 1 (*NAC*), PREDICTED: *C. sativus* NAC domain-containing protein 100 (*NAC3*), and PREDICTED: *C. sativus* hypothetical protein (*uk4*) was markedly upregulated by total defoliation, although the functions of these genes remain unclear (Tazuke et al., 2019). As described in Tazuke et al. (2019), varietal difference of the effect of total defoliation on the expression of the chosen 8 genes was examined. Cultivars used were as follows. Japanese type: ‘‘Tokiwa’, ‘High green 21’, and ‘Freedom house No. 2’; North-Chinese type: ‘Suyo’’; South-Chinese type: ‘Sagami hanshiro’;;Slicer:’Poinsett’; English greenhouse slicer: ‘Proloog RZ’; Beit Alpha ‘Khassib RZ’; Pickling: ‘Mogami’ together covering representative cucumber types of the world. When the fruit was about 9 cm long, total defoliation treatment was done at 9 a.m. and harvested 9 a.m. the next day. Although there was some varietal difference, in all cultivars, all the 8 genes were markedly upregulated (Tazuke et al., 2019). This suggested that the 8 genes may be good markers of sugar starvation for all the cucumber cultivars in the world.

Because microspore formation is sensitive to environmental conditions, such as moderate heat stress and low temperature (Picken, 1984; Sato et al., 2006; Mesihovic et al., 2016), parthenocarpy is a desired trait. Cucumber has naturally parthenocarpic cultivars. Many reports have shown that parthenocarpy is induced by plant hormones (Fu et al., 2008; Gorget et al., 2005; Serrani et al., 2010). Introduction of the indole-3-acetic acid (IAA)-synthesizing *DefH9-iaaM* gene in cucumber enhances parthenocarpy (Yin et al., 2006). In tomato, overexpression of the auxin (Aux) receptor *Solanum lycopersicum* Transport Inhibitor Response 1 (*SlTIR1*) gene (Ren et al., 2011) and suppression of expression of the *S. lycopersicum* Aux response factor 7 (*SlARF7*) gene (De Jong et al., 2009) and Aux/IAA *SlIAA9* gene (Wang et al., 2005) induce parthenocarpy. Parthenocarpy is also induced by gibberellin, and it is suggested that Aux works upstream of gibberellin (Serrani et al., 2010; Hu et al., 2018). However, the direct target of gibberellin is not known (Shinozaki et al., 2020). Kusano et al. (2022) performed transcriptomic, hormonomic, and metabolomic analyses of well-established tomato parhenocarpic mutants.

In cucumber, the results of the search for the genes responsible for parthenocarpy were equivocal. This may be partly attributed to differences in the measurements of parthenocarpy because Lietzow et al. (2016) used early growth as the measure of the extent of parthenocarpy, Wu et al. (2016) regarded fruits that did not abort as parthenocarpic, and Sun et al. (2006) used fruit yield as the measure of parthenocarpy. Therefore, additional observations of fruit growth and abortion are necessary to address this issue.

Cucumber fruits that fail to set eventually abort (Hikosaka and Sugiyama, 2004; Hikosaka and Sugiyama, 2005). It has been suggested that fruit abortion is caused by the source–sink relationship, which triggers sugar shortage in fruit (Marcelis, 1993). Thus, the investigation of sugar starvation in parthenocarpic fruit will be informative in this context. In addition to a genetic approach, understanding the physiological response of fruit set/abortion will be informative.

In this study, we observed the expression of candidate marker genes of sugar starvation in association with growth cessation in parthenocarpic cucumber fruit in a moderately parthenocarpic cultivar, as well as with fruit growth and sugar concentration to assess the physiological responses occurring in fruit set and abortion.

## 2 Materials and methods

### 2.1 Plant cultivation and growth analysis of fruits

Cucumber (*Cucumis sativus* L.) cv. ‘Tokiwa’ was used. It is a moderately parthenocarpic cultivar, suited to the study of parthenocarpy responses. Plants were grown in a glasshouse. Single plants were grown in 15 L containers filled with vermiculite. The plants were watered every morning with half-strength Hoagland No. 2 solution. Tap water was used to prepare the solution. The plants were detopped, leaving about 10 leaves. The lateral shoots were pinched, leaving the first node. The female flowers on the first nodes were used for the experiment. In the evening, female flower buds in which the petals had become yellow were judged to flower the next day and were wrapped with a paper bag to prevent pollination. The bag was removed on the day following the flowering. In the spring of 2019, fruit length was measured every morning until the fruit grew to marketable size or became soft indicating fruit abortion. In the spring of 2021, fruit length was measured every 2 days in the morning from the day of anthesis until 0, 2, 4, 6, and 8 days after anthesis (DAA), when the fruit was harvested. The relative growth rate in length of a fruit (RGR % h^–1^) was estimated as follows:


$$RGR=\frac{lnL_{1}-lnL_{0}}{24(d_{1}-d_{0})}\times 100,$$

where L_0_ and L_1_ are the lengths of the fruit at the beginning and end of the period of RGR measurement, respectively; and d_0_ and d_1_ are the dates of the beginning and end of the period, respectively. In 2019, one culture was done, and four cultures were done in 2021. In 2019, the growth curve was constructed until the fruits grew to marketable size or aborted. In 2021, the growth curve was constructed for fruits harvested on 8 DAA or for those that had aborted on 8 DAA or earlier combining the four cultures.

### 2.2 Fruit harvest

In the spring culture of 2021, fruits were harvested for gene expression and sugar analyses separately at 9 a.m. on 0, 2, 4, 6, and 8 DAA immediately after the length measurement. The harvested fruit was immediately weighed, placed in a jar filled with liquid nitrogen, and transferred to the laboratory. Samples for RNA extraction were stored at –80°C, if necessary, whereas samples for sugar extraction were stored at –30°C.

### 2.3 RNA extraction and reverse transcription

The frozen sample was powdered with a mortar and pestle and total RNA was extracted using the RNeasy Plant Mini Kit (QIAGEN, Hilden, Germany). DNA was removed with the RNase-Free DNase Set (QIAGEN). For reverse transcription, PrimeScript™ RT reagent Kit (Perfect Real Time) (TaKaRa, Kusatsu, Japan) was used. 2 mL total RNA was mixed with 2 mL Buffer, 0.5 mL enzyme, 0.5 mL oligo dT primer0.5 mL random 6 mersand and 4.5 mL RNase Free Water in a PCR tube and incubated at 37°C for 15 minutes and incubated at 85°C for 5 seconds to inactivate the enzyme then kept at 4°C with Eppendorf 5331 Mastercycler Gradient Thermal Cycler (Eppendorf Co. Ltd., Tokyo, Japan) and stored at –30°C.

### 2.4 RNA-seq and real-time PCR

The result of RNA-seq without biological replication (conducted by Hokkaido System Science Co. Ltd.) is shown in **Table S1**. Genes whose expression was enhanced by defoliation larger than 32 times are listed in Tazuke et al. (2019). The abbreviations of the name of genes are based on the description in GenBank. Expression of these genes were analyzed by real-time PCR, whose result is shown in Tazuke et al. (2019). From this result, 8 genes who showed large enhancement were selected as shown in **Table 1**. The primers used in this experiment were designed using Primer3web (https://primer3.ut.ee/). Selected reverse transcripts were amplified by PCR. Aliquots of the PCR products were subjected to electrophoresis to verify the specificity of the primers. The aliquots of the PCR products were subjected to a series of 10-fold dilutions with EASY Dilution (for real-time PCR) solution (TaKaRa) and used to produce the calibration curve. *C. sativus* clone CU36H1 actin was used as the internal standard. Two μL reverse transcript solution was mixed with 1 μL forward primer (10 μM), 1 μL reverse primer (10 μM), 12 μL Premix Ex Taq™ (Perfect Real Time; TaKaRa) and 4 μL sterilized water on a PCR plate. The PCR plate was set in a LightCycler^®^ 96 System (Roche, Basel, Switzerland). The cycle condition was 40 cycles of 95°C 5 seconds and 60°C 30 seconds.

**Table 1:** Descriptions of the genes subjected to real-time PCR.

Abbreviation	Gene name^z)^	Description in GenBank
*actin*	CsaV3_6G041900	*Cucumis sativus* clone CU36H1 actin mRNA, partial cds
*AS*	CsaV3_6G016090	PREDICTED: *Cucumis sativus* asparagine synthetase mRNA
*CsSEF1*	CsaV3_2G025800	*Cucumis sativus* zinc finger CCCH domain-containing protein 20
*CsFDI1*	CsaV3_7G023540	*Cucumis sativus* BTB/POZ domain-containing protein At1g63850-l like
*HB*	CsaV3_6G046050	PREDICTED: *Cucumis sativus* homeobox-leucine zipper protein ATHB-40
*PMS*	CsaV3_1G000860	PREDICTED: *Cucumis sativus* peptide methionine sulfoxide reductase
*NAC*	CsaV3_5G032930	PREDICTED: *Cucumis sativus* NAC domain-containing protein 1
*NA3*	CsaV3_3G041280	PREDICTED: *Cucumis sativus* NAC domain-containing protein 100
*uk4*	CsaV3_3G023510	PREDICTED: *Cucumis sativus* hypothetical protein

^z)^ Cucurbit Genomics Database Cucumber Chinese Long ver. 3.

### 2.5 Post harvest expression of the chosen 8 genes

Cucumber cultivar ‘Tokiwa’ was grown in a glasshouse in Spring of 2020 as described above. At 9 a.m., about 9 cm long pollinated young fruits were put into water vapor saturated boxes and transferred to the labolatory and the boxes were incubated at 20°C or 30°C. After 6, 12 and 24 hours of incubation, total RNA was extracted and analyzed by real-time PCR. For 0 hour, fruits harvested in the glasshouse was immediately put in a jar containing liquid nitrogen and transferred to the laboratory. Every treatment was triplicated.

### 2.6 Sugar analyses

The stored frozen samples were powdered with a mortar and pestle, homogenized with 80% ethanol, extracted for 15 min at 80°C, and filled up to 100 mL. Aliquots of 1 mL were dried with a rotary evaporator and redissolved in 1 mL water, and the concentrations of sucrose, glucose, and fructose were analyzed enzymatically with an F-kit (JK International Co. Ltd., Tokyo, Japan). Glucose and fructose are the major sugars in cucumber fruit (Handley et al., 1983). Here, we refer to the sum of the concentration of glucose and fructose as the hexose concentration. In cucumber fruit, sugar starvation must be hexose starvation.

### 2.7 Statistical analyses

The statistical analyses were performed using R (R Core Team, 2022). Correlation matrix was calculated by cor() function. P-value was calculated by cor.test() function. Multiple comparison was performed using TukeyHSD() and aov() functions. The data were log-transformed if appropriate. For the test of difference between average and constant, pt() function was used.

## 3 Results

### 3.1 Fruit growth

**Figure 1:**
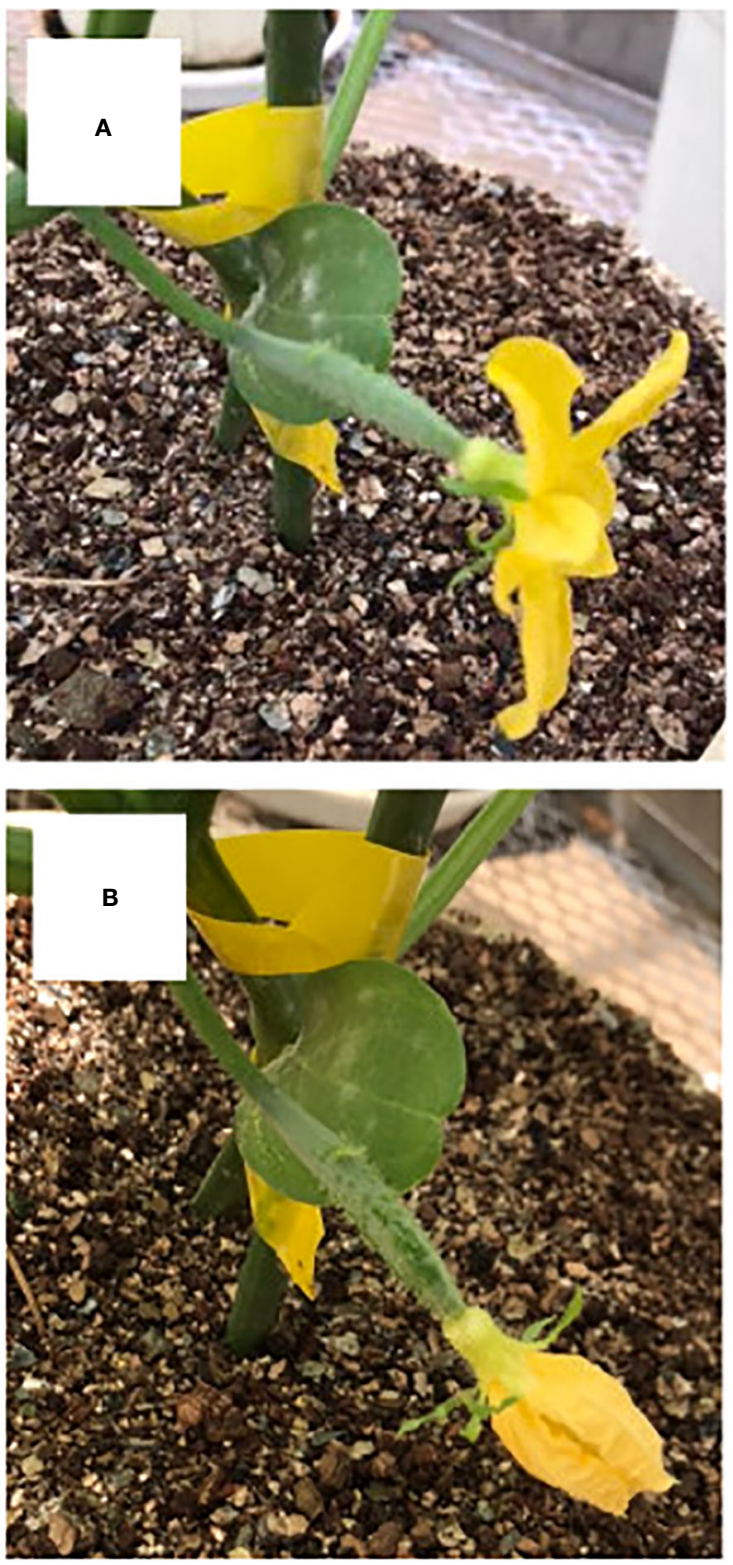
**(A)** Flower on the day of anthesis of parthenocarpic cucumber fruit. **(B)** Fruit 3 days after anthesis of parthenocarpic cucumber fruit.

**Figure 2:**
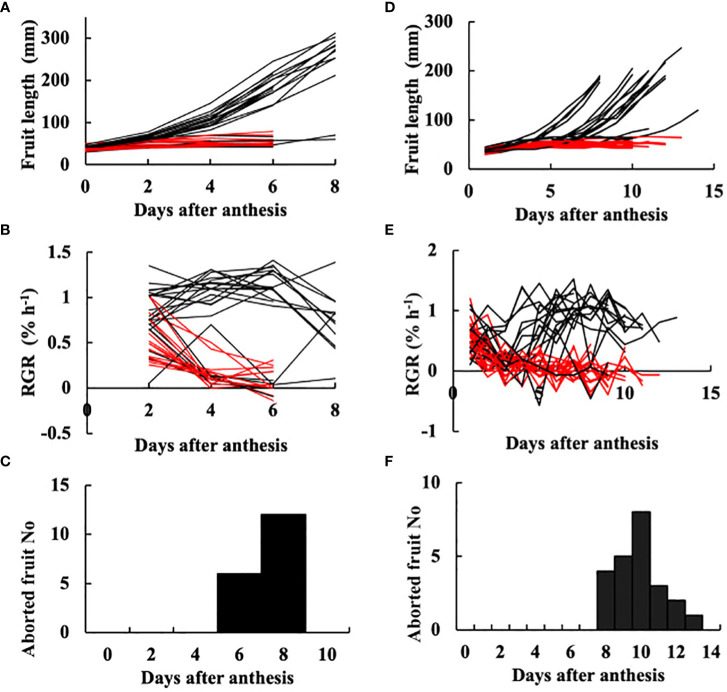
Growth of parthenocarpic cucumber fruit. **(A–C)** Experiment in 2021. **(D–F)** Experiment in 2019. **(A, D)** Growth in length plotted against days after anthesis (DAA). Each line corresponds to an individual fruit. **(B, E)** Relative growth rate (RGR) in fruit length. Each line corresponds to an individual fruit. **(C, F)** Histogram of the date of fruit abortion. **(A, B, D, E)** Red lines indicate individual fruits that eventually aborted. Black lines indicate fruits that did not abort.

Fruit on the day of anthesis is shown in **Figure 1A**. Fruit at 3 DAA is shown in **Figure 1B**, at which time point they had not begun exponential growth, which is a typical symptom of growth suppression of parthenocarpic fruit. The growth of fruit based on length is shown in **Figures 2A, D**. RGR in fruit length is shown in **Figures 2B, E**. In the growth analysis of 2021 (**Figures 2A, B**), the growth of most fruit could be classified into those that eventually aborted and those that did not abort. Among the 41 fruits examined, 18 aborted yielding an abortion rate of 43.9%. The date of fruit abortion was concentrated on 6 DAA and 8 DAA (**Figure 2C**). In the growth analysis of 2019, the date of onset of exponential growth was variable (**Figures 2D, E**). Most of the fruits belonged to one of two classes: those that grew to marketable size and those that eventually aborted. Fruits with low growth activity eventually aborted, but the date of abortion was variable (**Figure 2F**). In the 41 examined fruits, 23 aborted yielding an abortion rate of 56.1%. These abortion rates verified that ‘Tokiwa’ is an intermediately parthenocarpic cultivar, which confirms that it is suited for analysis of the parthenocarpic traits.

### 3.2 Evaluation of the expression of genes related to sugar signaling

#### 3.2.1 Hexokinase 1

In *Arabidopsis*, hexokinase 1 (HXK1) is a well-known sugar sensor. So, we sought in the **Table S1** for the keyword “hexokinase 1”. One hit was obtained: Csa2G000870. Because the RNA-seq was analyzed using Chinese Long ver. 2, the gene code is different from that of Chinese long ver. 3. The enhancement of the expression was less than 2.

#### 3.2.2 SnRK1

Luo et al. (2022) have done genome-wide analysis of *SnRK* gene family in cucumber. They found CsaV3_6G006250 was homologous to *AtSnRK1*. This gene corresponds to Csa6G077450 in Chinese Long ver. 2. However, this gene was not found in **Table S1**. So, *Arabidopsis* protein sequence AT3G01090’s homolog was sought in Chinese Long ver. 2 database. Csa6G077450 had E-value 0, but this gene could not be found in **Table S1**. Csa4G269780 had E-value e-92. This gene’s expression enhancement was about 2.

#### 3.2.3 TOR

The protein sequence of *AtTORC1*: AT1G50030’s homolog was sought by BLAST in Chinese long ver. 2 database. Csa7G070760 was obtained. Its enhancement of expression was about 2.

### 3.3 Gene expression of fruits without pollination

**Figure 3:**
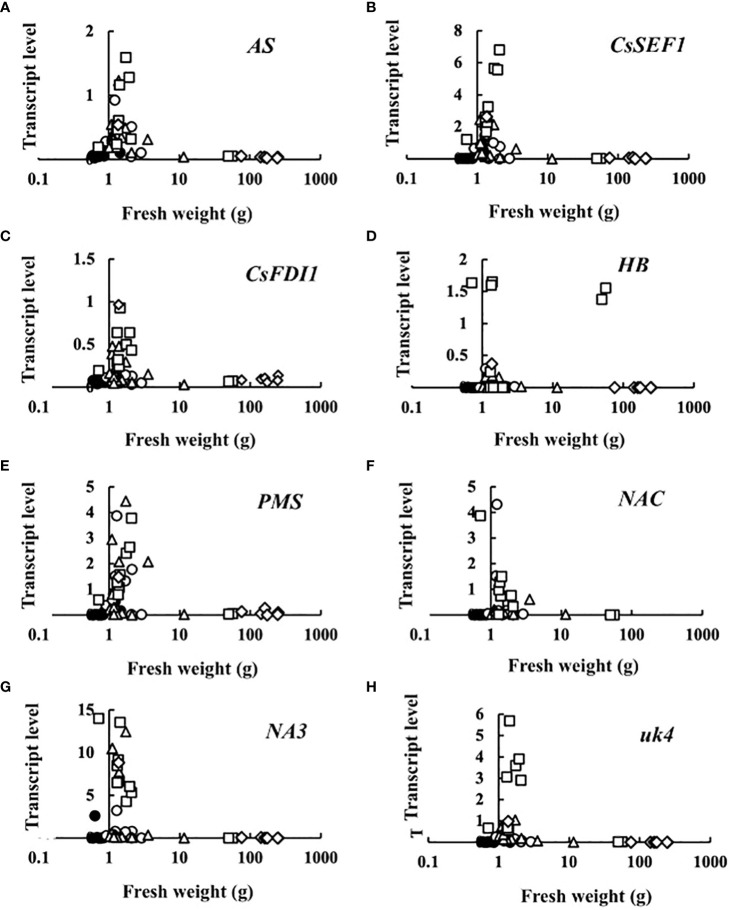
Relationship between fruit fresh weight and transcript levels of **(A)** asparagine synthetase (*AS*), **(B)** *Cucumis sativus* putative CCCH-type zinc finger protein CsSEF1 (*CsSEF1*), **(C)** *C. sativus* BTB/POZ domain-containing protein At1g63850-like (*CsFDI1*), **(D)** *C. sativus* homeobox-leucine zipper protein ATHB-40 (*HB*), **(E)** *C. sativus* peptide methionine sulfoxide reductase (*PMS*), **(F)** *C. sativus* NAC domain-containing protein 1 (*NAC*), **(G)** *C. sativus* NAC domain-containing protein 100 (*NA3*), and **(H)** *C. sativus* hypothetical protein (*uk4*) of parthenocarpic cucumber fruit. •: The day of anthesis, ○: 2 days after anthesis (DAA), △: 4 DAA, □: 6 DAA, ◇: 8 DAA. Description of the genes is given in **Table 1**.

Transcript levels plotted against fruit fresh weight are shown in **Figure 3**. At 0 DAA, the transcript levels of all eight genes were very small. The increase in transcript levels was restricted to very small fruits, suggesting that sugar starvation only occurred in growth-suppressed fruit. An exception was *HB* in which some fruits showed high transcript levels at a relatively high fresh weight.

**Figure 4:**
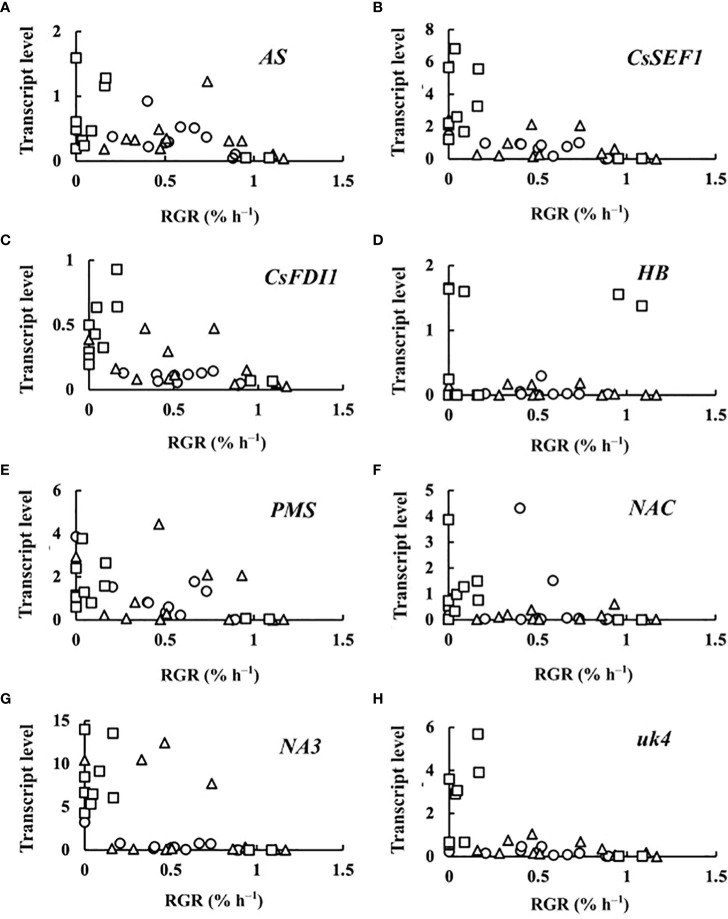
Relationship between relative growth rate (RGR) in fruit length and transcript levels of **(A)** asparagine synthetase (*AS*), **(B)** *Cucumis sativus* putative CCCH-type zinc finger protein CsSEF1 (*CsSEF1*), **(C)** *C. sativus* BTB/POZ domain-containing protein At1g63850-like (*CsFDI1*), **(D)** *C. sativus* homeobox-leucine zipper protein ATHB-40 (*HB*), **(E)** *C. sativus* peptide methionine sulfoxide reductase (*PMS*), **(F)** *C. sativus* NAC domain-containing protein 1 (*NAC*), **(G)** *C. sativus* NAC domain-containing protein 100 (*NA3*), and **(H)** *C. sativus* hypothetical protein (*uk4*) of parthenocarpic cucumber fruit. ○: 2 days after anthesis (DAA), △: 4 DAA, □: 6 DAA, ◇: 8 DAA. Description of the genes is given in **Table 1**.

The transcript levels plotted against RGR are shown in **Figure 4**. Because RGR could not be calculated for 0 DAA fruits, the relationship between RGR and transcript levels was examined at 2, 4, 6, and 8 DAA. The transcript levels were mostly close to zero but tended to become high in fruits whose RGRs became close to zero. The correlation matrix of RGR and transcript levels are shown in **Table 2**. All of the correlation coefficients, except those for *HB* and *NAC*, were highly significant. For *HB*, the absolute values of the correlation coefficient were very low and insignificant. For *NAC*, correlation coefficients were insignificant or poorly significant. Negative correlation coefficients between RGR and transcript levels indicate that transcript levels become higher at lower RGRs. Highly significant positive correlation coefficients between transcript levels of *AS*, *CsSEF1*, *CsFDI1*, *PMS*, *NA3*, and *uk4* indicate that these genes are responding similarly to fruit growth.

**Table 2:** Correlation matrix of relative growth rate (RGR) in fruit length and transcript levels of candidate marker genes of fruit sugar starvation in cucumber.

	*AS*	*CsSEF1*	*CsFDI1*	*HB*	*PMS*	*NAC*	*NA3*	*uk4*
(a) Correlation matrix NS: p > 0.05; *p < 0.05; **p < 0.01; ***p <0.001								
*RGR*	–0.4722**	–0.6198***	–0.5760***	–0.1336NS	–0.4962***	–0.3501*	–0.6100***	–0.4863**
*AS*		0.6968***	0.6299***	–0.0679NS	0.5419***	0.3172NS	0.4581**	0.6622***
*CsSEF1*			0.7214***	–0.0120NS	0.7249***	0.1844NS	0.5611***	0.8123***
*CsFDI1*				–0.0018NS	0.5027***	0.2977NS	0.7348***	0.7909***
*HB*					–0.1065NS	0.3046NS	0.3285*	–0.1004NS
*PMS*						0.0584NS	0.542***	0.4686**
*NAC*							0.3907*	0.2197NS
*NA3*								0.5527***
(b) *p*-value matrix								
*RGR*	0.0018	1.6E–05	8.1E–05	0.405	9.7E–04	0.0248	2.3E–05	0.0013
*AS*		4.2E–07	1.0E–05	0.6733	2.5E–04	0.0433	0.0026	2.4E–06
*CsSEF1*			1.0E–07	0.9405	8.4E–08	0.2483	1.4E–04	1.1E–10
*CsFDI1*				0.9912	8.1E–04	0.0587	4.5E–08	7.6E–10
*HB*					0.5076	0.0528	0.036	0.5321
*PMS*						0.7167	2.5E–04	0.002
*NAC*							0.0116	0.1675
*NA3*								1.8E–04

NS, not significant.

### 3.4 Sugar levels

**Figure 5:**
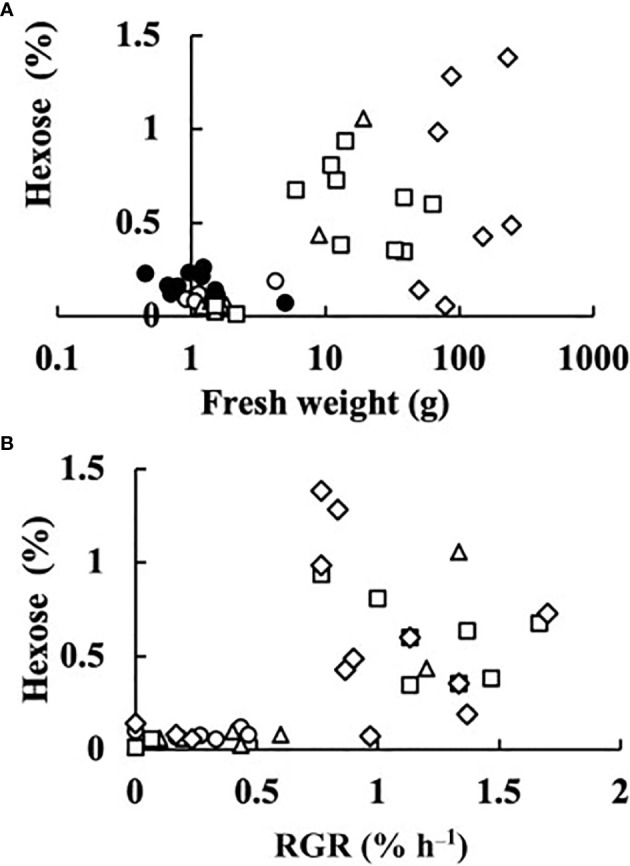
Dependence of hexose (glucose + fructose) concentration of parthenocarpic cucumber fruits on growth parameters. •: The day of anthesis, ○: 2 days after anthesis (DAA), △: 4 DAA, □: 6 DAA, ◇: 8 DAA. **(A)** Fruit fresh weight. **(B)** Relative growth rate (RGR) in fruit length.

**Figure 6:**
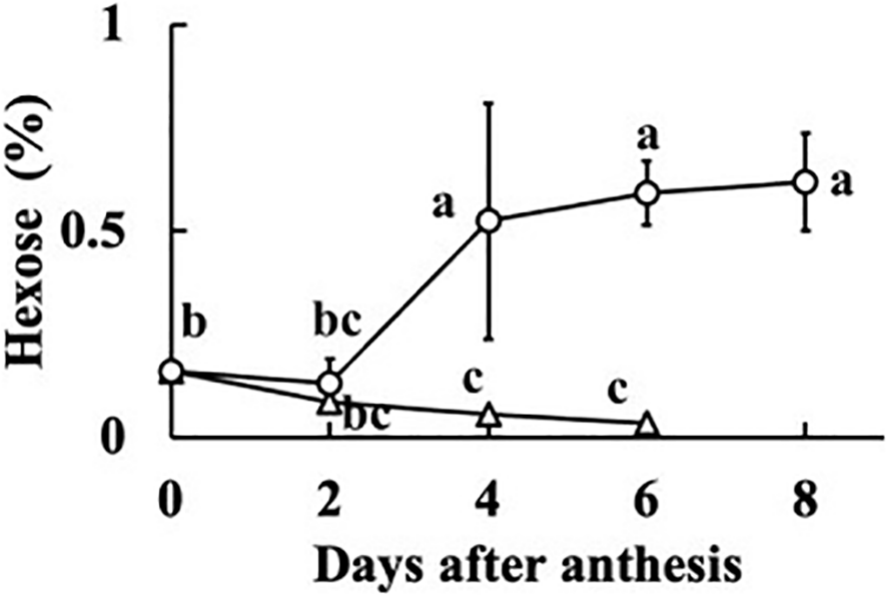
Change in hexose concentration of growth-suppressed (relative growth rate [RGR] < 0.5% h^–1^) and not growth-suppressed (RGR > 0.5% h^–1^) parthenocarpic cucumber fruit. Vertical bars represent the standard error of the mean. Different letters indicate 5% significant difference.

In all fruits, the sucrose concentration was very low, and did not differ much from zero (*p =* 0.044). The sum of the concentrations of glucose and fructose was regarded as the hexose concentration. The relationships between fruit fresh weight or RGR and hexose concentration at 0, 2, 4, 6, and 8 DAA are shown in **Figure 5**. At 0 DAA, the hexose concentration was very low but the average was significantly larger than zero (*p* = 1.18E–05). In fruits whose growth was suppressed, as seen from the low values of fresh weight and RGR, hexose concentration was very low. In fruits that grew normally, hexose concentration was high. The correlation coefficient between fruit fresh weight and hexose concentration was 0.5336 and the *p*-value was 9.4E–05 (*n* = 48). The correlation coefficient between RGR and hexose concentration was 0.5476, and the *p*-value was 0.00012 (*n* = 37). The relationship between hexose concentration and RGR was remarkable. There was a clear transition from low to high hexose concentration around RGR = 0.5% h^–1^ (**Figure 5B**). Under salinity, fruit RGR declined to 0.5% h^–1^ (Tazuke, 1997). So, the average of all data of 0 DAA and the average of data for which RGR was lower than 0.5% h^–1^ after 2 DAA were plotted. Two fruits at 8 DAA that had low RGR but had relatively high fresh weight were excluded. Averages of hexose concentration of fruits whose RGR was larger than 0.5% h^–1^ were also plotted (**Figure 6**). Hexose concentration of growth-suppressed fruits decreased up to 6 DAA toward zero.

### 3.5 Postharvest gene expression

**Figure 7:**
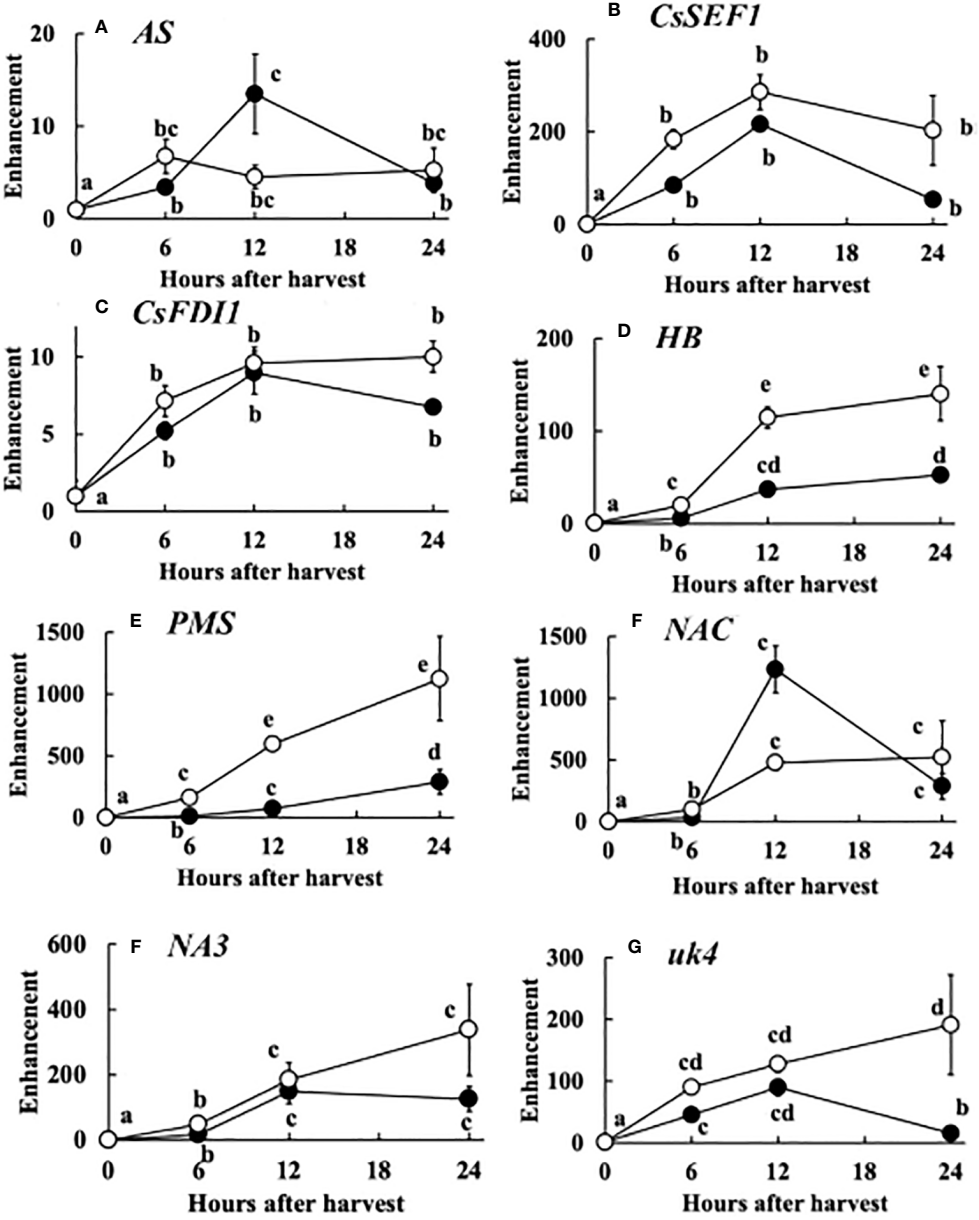
Post-harvest change in the expression of 8 genes, i.e. **(A)** *AS*, **(B)** *CsSEF1*, **(C)** *CsFDI1*, **(D)** *HB*, **(E)** *PMS*, **(F)** *NAC*, **(G)** *NA3*, **(H)** *uk4*. The meanings of the abbreviations are shown in **Table 1**. Harvested cucumber fruits were incubated at 20°C (closed circles) or 30°C (open circles). Vertical bars are SE of means. Different letters indicate 5% significant difference.

The expression of the 8 genes increased up to 12 hours after harvest. However, in many genes especially at 20°C, the expression decreased at 24 hours after harvest. The enhancement of the expression of each gene compared to that at the harvest was similar to total defoliation treatment (**Figure 7**).

## 4 Discussion

As seen from RNA-seq data (**Table S1**), the enhancement of the expression of well-known sugar signaling genes, HXK1, SnRK1 and TOR was not much larger than 2. This may mean that these genes are constantly expressed and deliver the signal to the genes at lower cascade. Therefore, these genes are not suited for the marker of sugar starvation.

Hexose concentration was low at 0 DAA and increased in fruits with high RGR, reflecting exponential growth. This is consistent with vacuolization with the accumulation of hexose in the vacuole in normally growing fruit (Shinozaki et al., 2020). The hexose concentration at 0 DAA was very low but was significantly higher than zero. From this stage, fruit can grow normally, meaning that this low hexose concentration may be sufficient. At this stage, the vacuole may not have developed much, and this hexose concentration may therefore reflect that of cytosol. Hexose concentration in growth-suppressed fruit decreased until 6 DAA toward zero. In about 9 cm cucumber fruit, total defoliation and prolonged darkness enhanced the expression of *AS* and *CsSEF1*, but hexose concentration was not much affected by the treatment (Tazuke and Asayama, 2013), possibly because most hexose was in the vacuole. On the other hand, the close-to-zero hexose concentration seen in growth-suppressed parthenocarpic fruit may reflect little vacuolization. In this condition, explanation of the expression of candidate marker genes will be very influential. Sucrose concentration in all of the fruits examined was very close to zero. In most plants that translocate sucrose, a sucrose concentration gradient is often supposed to determine the sink activity (Ho, 1988; Ho, 1996). However, the main translocated sugars are stachyose and raffinose in cucumber (Turgeon and Wolf, 2009; Dai et al., 2023). Whether the gradient of these sugars is related to sink activity in cucumber fruit remains to be investigated.

For *AS*, *CsSEF1*, and *CsFDI1*, correlation of their transcript levels with each other was positive and highly significant, and with RGR, the correlation was negative and highly significant. In these three genes, the upregulation of the genes was mainly seen on 6 DAA. Because the expression of these three genes was tested in many experiments (Tazuke and Asayama, 2013; Tazuke et al., 2015; Tazuke et al., 2017; Tazuke and Kinoshita, 2018; Tazuke et al., 2019), it is highly likely that they are good markers of sugar starvation, although the gene function is known only in *AS*. Thus, in fruits whose RGR became close to zero, it is most probable that severe sugar starvation is occurring. The upregulation of candidate sugar starvation genes together with the reduction of hexose concentration close to zero strongly suggests that the candidate genes are genuine sugar starvation markers. In an experiment removing the ovary, it was suggested that stopping of sugar import (loss of sink activity) together with the remaining growth activity causes sugar starvation (Tazuke et al., 2017). Thus, loss of sink activity may occur in slow growing fruits, and it is possible to hypothesize that sugar starvation caused by loss of sink activity triggers fruit abortion. In the growth analysis of 2019, DAA of the onset of exponential growth was variable (**Figures 2D, E**), which indicates that the use of initial growth rate as the indicator of parthenocarpy (Lietzow et al., 2016) is inappropriate. The source–sink relationship and environmental factors reportedly affect the onset of exponential fruit growth (Marcelis, 1993; Hikosaka and Sugiyama, 2004; Hikosaka and Sugiyama, 2005). Because the suppression of fruit growth and fruit abortion that follows are affected by the source–sink relationship and environmental factors, the use of fruit abortion as the indicator of parthenocarpy (Wu et al., 2016) may lead to confusion, which is also the case in the use of fruit yield (Sun et al., 2006).

For newly identified genes by RNA-seq (Tazuke et al., 2019), *PMS*, *NA3*, and *uk4* showed a highly significant negative correlation with RGR and a highly significant positive correlation with the transcript levels of *AS*, *CsSEF1*, and *CsFDI1*. They also showed a highly significant positive correlation among their transcript levels. Thus, it is likely that *PMS*, *NA3*, and *uk4* are also good marker genes of sugar starvation. For the other two genes, *HB* and *NAC*, correlation matrix elements were mostly insignificant. In *HB*, the relationship with fruit fresh weight was abnormal. In *NAC*, outliers of the relationship between RGR and transcript levels were seen, which might have caused the insignificant correlation. The lack of correlation in *HB* and *NAC* is interesting. Both *HB* and *NAC* responded to total defoliation as examined by RNA-seq and real-time PCR (Tazuke et al., 2019). It is therefore possible that they respond to some signal under total defoliation other than sugar starvation. The investigation of *HB* and *NAC* may reveal unidentified physiological responses under total defoliation.

The post-harvest gene expression was similar to that of total defoliation except enhancement was much larger in post-harvest (**Figure 7**). This suggest that drastic sugar starvation is occurring after harvest. Therefore, these 8 genes can be good tools for the study of post-harvest physiology.

In sum, hexose measurement and expression of candidate marker genes together conclusively suggest that severe sugar starvation is occurring in growth-suppressed parthenocarpic cucumber fruit. As suggested by Shinozaki et al. (2020), hexose accumulation and vacuolization may be critical to the fruit set. How the sink activity is regulated to import sugars needs further investigations. The effects of total defoliation, parthenocarpy and post-harvest on the expression of the 6 genes were similar, but there were some differences. These differences may suggest the physiological status of fruits under these treatments. Prolonged failure of the onset of fruit growth eventually cause fruit abortion. Sugar starvation triggers autophagy (van Rensburg et al., 2019). So, it is possible that fruit abortion is caused by autophagy. Six out of eight candidate genes were suggested to be valid. These genes may be sensitive sensors of sugar starvation even in well-vacuolated tissue and can contribute to the study of stress physiology. Analyses of expression from the gene promoters may contribute to clarifying the signal transduction mechanism under sugar starvation. The remaining two genes seemed to respond to different stimuli.

## Data availability statement

The datasets presented in this study can be found in online repositories. The names of the repository/repositories and accession number(s) can be found in the article/**Supplementary Material**.

## Author contributions

AT planned and supervised the experiment, MA helped in molecular techniques and joined discussion. TK helped in statistical analyses. All authors contributed to the article and approved the submitted version.

## Supplementary material

Supplementary Table 1 Analysis of RNA-seq data.
